# Efficient Hole Trapping in Carbon Dot/Oxygen‐Modified Carbon Nitride Heterojunction Photocatalysts for Enhanced Methanol Production from CO_2_ under Neutral Conditions

**DOI:** 10.1002/anie.202105570

**Published:** 2021-08-24

**Authors:** Yiou Wang, Robert Godin, James R. Durrant, Junwang Tang

**Affiliations:** ^1^ Department of Chemical Engineering UCL Torrington Place London WC1E 7JE UK; ^2^ Department of Chemistry and Center for Plastic Electronics Imperial College London Exhibition Road London SW7 2AZ UK; ^3^ Department of Chemistry The University of British Columbia Kelowna BC V1V 1V7 Canada; ^4^ Chair for Photonics and Optoelectronics, Nano-Institute Munich Ludwig-Maximilians-Universität München Königinstr. 10 80539 Munich Germany

**Keywords:** carbon dioxide fixation, carbon dots, charge trapping, photocatalysis, time-resolved spectroscopy

## Abstract

Artificial photosynthesis of alcohols from CO_2_ is still unsatisfactory owing to the rapid charge relaxation compared to the sluggish photoreactions and the oxidation of alcohol products. Here, we demonstrate that CO_2_ is reduced to methanol with 100 % selectivity using water as the only electron donor on a carbon nitride‐like polymer (FAT) decorated with carbon dots. The quantum efficiency of 5.9 % (*λ*=420 nm) is 300 % higher than the previously reported carbon nitride junction. Using transient absorption spectroscopy, we observed that holes in FAT could be extracted by the carbon dots with nearly 75 % efficiency before they become unreactive by trapping. Extraction of holes resulted in a greater density of photoelectrons, indicative of reduced recombination of shorter‐lived reactive electrons. This work offers a strategy to promote photocatalysis by increasing the amount of reactive photogenerated charges via structure engineering and extraction before energy losses by deep trapping.

## Graphical abstract

A modified carbon nitride with O‐containing linkers decorated with carbon dots selectively produces methanol from CO_2_ and water with the benchmark quantum yield of ca. 6 %. Transient absorption spectroscopic investigation shows that extraction of holes before trapping by the carbon dots is key for the remarkable performance.

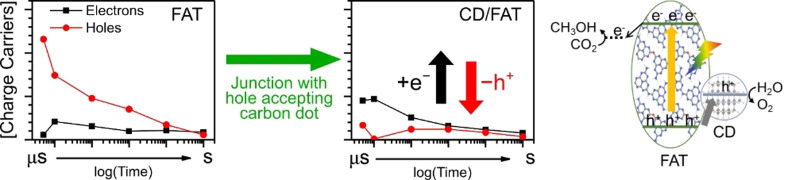

## Introduction

Natural photosynthesis provides sustainable energy for life and maintains an ecological balance on the planet by fixing CO_2_ into organics.^[1]^ Continuous efforts have been put into economic artificial photosynthesis to convert CO_2_ using inexhaustible solar energy.^[2]^ The efficiency of CO_2_ reduction has been improved through different routes over the last decades, including the photoelectrochemical processes,^[3]^ the optimisation of cocatalysts,^[4]^ and the fabrication of junction photocatalysts.^[5]^ These processes use either expensive and unsustainable sacrificial reagents or abundant water as electron/proton donors.^[6]^ The latter involves CO_2_ reduction by photoelectrons coupled with water oxidation by photoholes, competing with the typically much faster charge recombination and the reduction of protons to H_2_. Therefore, it is much more challenging to couple CO_2_ fixation with pure water oxidation.

The intrinsic properties of a photocatalyst are crucial for photon‐driven chemical processes. Carbon nitride (CN) is one of the most promising metal‐free photocatalysts due to its high visible‐light‐driven performance for hydrogen production and CO_2_ reduction.^[7]^ However, rapid charge recombination and limited visible light absorption due to its wide band gap (2.7–2.9 eV) still limit its performance.^[8]^ It is crucial to optimise the photocatalytic activity of CN by reducing such severe charge recombination and enhance the light absorption via the novel design of materials.^[9]^

The engineering of the linker/terminal groups is believed to be a promising route to improve the efficiency of polymeric photocatalysts.[^7b^, ^10^] As previously reported, nitrogen linkers/terminals can be replaced by oxygen in CN.^[7b]^ Thus, the band positions, band gaps, and hydrophilicity of heptazine‐based semiconductors can be easily tuned to stepwise enhance the charge separation, visible‐light absorption, and the contact with water. The superior properties resulted in an enhancement of ca. 20 times in hydrogen and oxygen production from water compared with CN.[^10c^, ^11^] The lifetime of photoelectrons is more demanding in the reduction of CO_2_ to methanol, a six‐electron process, compared to a two‐electron process of H_2_ production in water splitting. Interestingly, the carbon‐dot (CD) as a selective hole acceptor was very recently reported by us, resulting in photoelectrons with an extended lifetime.^[6a]^

Herein, using transient absorption spectroscopy (TAS), we distinguish the nature of charge trapping in a linker‐controlled polymer (FAT) synthesised from formic‐acid‐treated dicyandiamide from that of pristine CN. We find that CN mainly traps electrons while FAT mainly traps holes based on the amplitudes of the deconvoluted components. Coupling this FAT polymer with efficient hole‐accepting CD led to selective CO_2_ reduction to methanol by water under neutral conditions. O_2_ is the only oxidation product and is produced in a near stoichiometry ratio. The reaction proceeds with nearly 6 % internal quantum yield (IQY) at 420 nm, which sets a new benchmark for metal‐free systems and is superior to metal‐containing systems (Table S1).^[6a]^ The change to fast hole trapping in FAT is key since the CD can subsequently extract holes on the sub‐microsecond timescale. With CN, trapped electrons accumulate, which increases the charge carrier density and accelerates the rate of recombination, leading to a lower activity under continuous irradiation according to our previous report.^[12]^ The CD/FAT system shows how selective extraction of holes with a propensity for trapping can lead to significant improvements in activity. This novel strategy to promote both internal charge separation and to incorporate an efficient hole acceptor provides an efficient approach to artificial photosynthesis, thus paving a sustainable and clean avenue to economic CO_2_ conversion and solar energy storage using metal‐free materials.

## Results and Discussion

**Figure 1:**
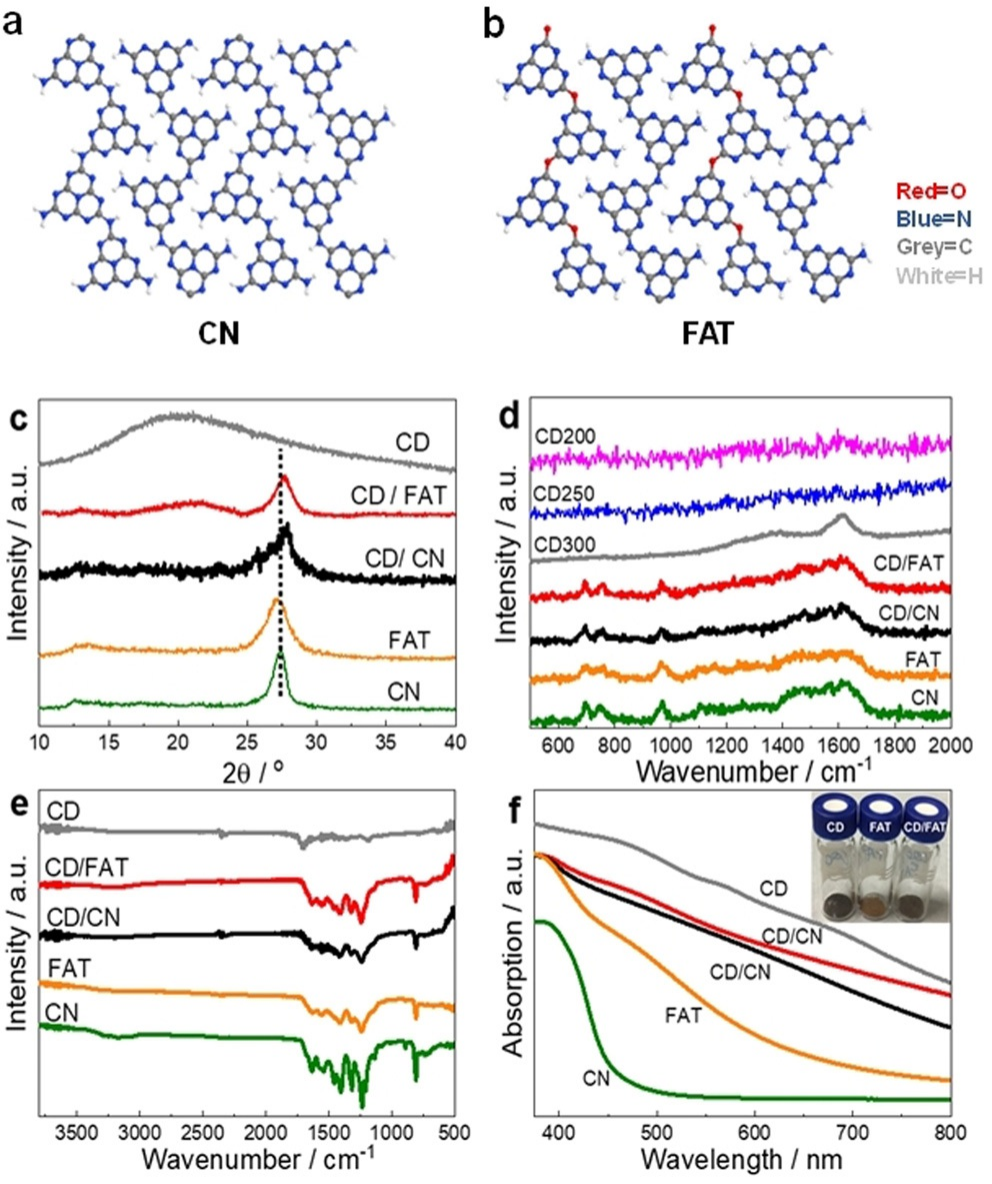
Structure of a) CN and b) FAT polymers. Characterizations of CD, CN, FAT, CD/CN and CD/FAT. c) PXRD pattern, d) Raman, e) FTIR and f) UV/Vis spectra. Inset: image of CD, FAT and CD/FAT.

Treating dicyandiamide by formic acid creates an oxygen‐containing precursor,[^6a^, ^13^] which is then heated to produce FAT with oxygen‐ and nitrogen‐linked heptazine‐domains (Figure 1 a and b).^[13]^ CN, as a reference, was synthesised from dicyandiamide. The efficient CD cocatalyst was prepared from citric acid and urea via a microwave‐assisted method.^[6a]^ The CD samples synthesised under different synthetic microwave powers are denoted as CDx, where *x* stands for the power in W. When not specified, CD refers to CD300. The CD was loaded on FAT and CN by suspending CD with the polymer in 10 mL DMF, drying and annealing the samples again at 500 °C for four hours. Detailed preparation procedures and other characterisation methods are listed in the Electronic Supporting Information (ESI).

Firstly, thorough characterisations have been carried out to investigate the structure and composition of all photocatalysts. According to the X‐ray diffraction (XRD) patterns (Figure 1 c), FAT and CN exhibit the typical diffraction signals of (002) and (100) at 27.1° (3.29 Å), 13.3° (6.65 Å) and 27.4° (3.26 Å), 13.0° (6.82 Å), respectively.[^7b^, ^10c^] The shifts of FAT from pristine CN are due to more distorted structure and smaller size of unit cells.^[10c]^ CD shows a broadened (002) band near that of graphene reported in the literature (26.5°, 3.36 Å), indicating a graphene‐derived structure. Surprisingly, after coupling the polymers with CD to form junctions, the interlayer distances of both CD/FAT and CD/CN have been compacted to 3.20 Å (27.8°), suggesting the formed junction possesses stronger interaction and attraction. Electronic effects might reduce the repulsion between layers and potentially indicates some perpendicular arrangement between CD and polymer. Reduction of the interplanar stacking distance has been linked to increased mobility in CN^[14]^ and may contribute to the enhanced activity (*vide infra*).

Raman spectroscopy was used to examine the backbone of materials (Figure 1 d). Characteristic vibrations of the heptazine‐based 2D structure have been found on FAT, CN, CD/FAT and CD/CN, with the peaks in the regions of 1200–1700 cm^−1^, 980 cm^−1^ and 690 cm^−1^ corresponding to the disordered graphitic carbon‐nitrogen vibrations, the symmetric N‐breathing mode of heptazine and the in‐plane bending, respectively.^[15]^ For CD synthesised at different microwave powers, CD200 and CD250 could not show distinct graphitic signals. Only CD300/FAT has a clear graphitic structure of D‐band around 1350 cm^−1^ and G‐band around 1600 cm^−1^, implying the CD300 still maintains a relatively integrated sp^2^ structure of graphene even after forming the heterojunction.

Fourier‐transform infrared spectroscopy (FTIR) was carried out to identify the assignment of the proposed structure (Figure 1 e). FAT shows similar trends to CN except that signals related to C‐NH_*x*_ at 1200 cm^−1^ and 1450 cm^−1^ are weaker, and the peaks of NH_*x*_ around 3000–3300 cm^−1^ change to a broad band of O.^[10c]^ Besides C−C vibrations at 1200 cm^−1^ and 1450 cm^−1^, the CD also displays weak signals around 1250–1550 cm^−1^ of C‐N vibrations and a peak of C=O around 1730 cm^−1^, suggesting the incorporation of N and O atoms. As for CD/FAT and CD/CN, one can still see the less pronounced C‐N vibrations of CD (1250–1550 cm^−1^), which are different from heptazine characters, confirming the co‐existence of CD and polymers. C=O peak in CD disappeared in the junctions likely due to their reaction during the annealing. The HRTEM image of CD in CD/FAT nanocomposite (Figure S1) further confirms the coexistence of CD and the polymer, and the CD in our study is crystallised graphite phase, consistent with Raman spectra in Figure 1 d. One can observe the regions of fringes with a characteristic d‐spacing of 0.32 nm across the sample, which agrees with the (002) values calculated from XRD patterns of CD/FAT. Based on the previous observation,^[12]^ the crystal structure of CN was relatively more sensitive to the electron beam irradiation and was therefore damaged after a short time observation, while that of CD remained intact. Such regions of graphitic fringes are thus attributed to CD.

According to UV/Vis spectroscopy, the brownish CD powder (Figure 1 f and inset) absorbs light from UV to the near IR. X‐ray photoelectron spectroscopy (XPS) was used to identify different elements (C, N, O) and the chemical states in CD, FAT, CN and their junctions (Figure S2,S3, Table S2–S4). XPS survey spectrum of CD shows its surface chemical composition of at. 11.86 % N, 61.99 % C, and 26.15 % O. Peaks of C‐NH_*x*_, C‐OH, and C=O detected in CD confirm a large portion of the N/O dopants are located outside the graphitic framework and are present as surface groups, correlating with the proposed sp^2^ graphene structure from Raman spectroscopy.^[16]^ Obvious C=O signals are only observed in CD, which agrees with the FTIR spectrum. The compositions of C and N states are similar to those previously reported.^[10c]^ The O 1s XPS spectrum indicates that all samples have C‐OH on the surface, which favours the intimate interaction between CD and polymer layers during junction fabrication due to hydrogen bonds and increases hydrophilicity to promote the contact with water.^[16]^

**Figure 2:**
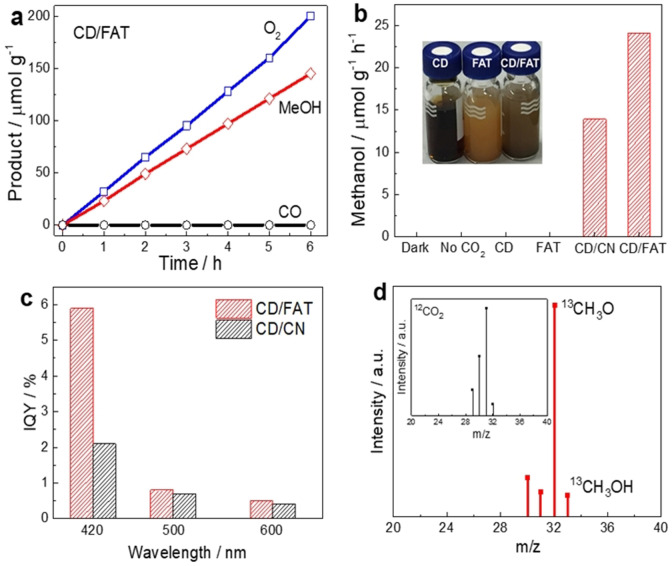
Photocatalytic CO_2_ conversion to methanol. The photocatalytic activity of a) CD/FAT measured under visible light (*λ*>420 nm). b) Control experiments on CD, FAT, CD/CN. Inset: image of CD, FAT, and CD/FAT. c) IQY of CD/FAT and CD/CN measured at atmospheric pressure under nearly one sun irradiation condition. d) Mass spectra of the product ^13^CH_3_OH from ^13^CO_2_ photoconversion by the CD/FAT photocatalyst.

**Figure 3:**
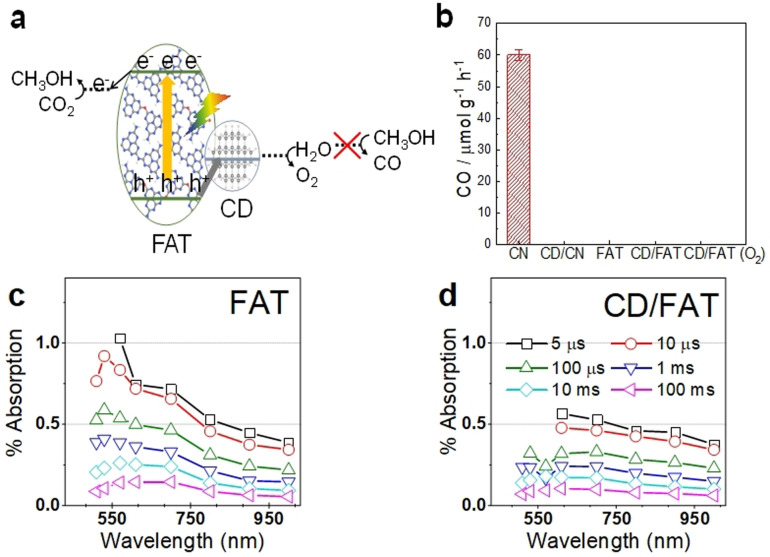
a) The CD/FAT junction. b) Methanol oxidation test on CD, CD/CN, FAT and CD/FAT. TAS measurements of FAT and CD/FAT. Diffuse reflectance TAS spectra for c) FAT and d) CD/FAT.

After understanding the structure of synthesised photocatalysts, their performance for artificial photosynthesis was evaluated at 1 bar condition under irradiation of 300 W Xenon light source with a light intensity of 100 mW cm^−2^ (420 nm<*λ*<710 nm) to simulate practical environmental conditions. Methanol is selectively produced from CO_2_ and water at a rate of 24.2 μmol g^−1^ h^−1^ on CD/FAT at neutral pH (Figure 2 a). Moreover, the methanol:O_2_ product ratio of 2:2.64 is close to the expected 2:3 stoichiometry of formula 2 CO_2_ + 4 H_2_O → 2 CH_3_OH + 3 O_2_. The reason for the smaller amount of O_2_ might be attributed to some side reactions related to O_2_. No product of CO from methanol oxidation (by O_2_ or photoholes) could be detected (Figure 3 b), indicating the surface of FAT and CD/FAT is favourable to the selective reduction of CO_2_ instead of the back reaction of methanol oxidation. In comparison, CD/CN shows two thirds of the activity for methanol production at a rate of 13.9 μmol g^−1^ h^−1^ (Figure 2 b). Control experiments showed that no product such as methanol or CO was detected under dark conditions, with only argon as the feed gas or uing solely CD or FAT photocatalysts (Figure 2 b).

Carbon dots are usually a mixture of many structures.^[17]^ To identify the possible functional structure of the carbon dots in our study, we carried out the photocatalytic CO_2_‐to‐methanol on FAT decorated with CD synthesised under different microwave powers (CD200, CD250, CD300 for 200, 250, 300 W, respectively; Figure 1 b). We observe that the trend in activity (Figure S5a) agrees with the degree of crystallisation in the Raman spectra and HRTEM. It indicates that more crystallised graphite structure is favourable to the photocatalytic performance. The similarity of 2D structure between FAT and CD benefits the stacking of layers as noted in the reduced layer distance in FAT, which enhances the contact between interfaces for charge transfer. The weight ratio between CD and FAT has been optimised to be 10 % (Figure S5b) since a higher concentration could result in the shielding of light. The starting pH of the system influences the state of CO_2_ in water. In this study, CD/FAT junction works stably under neutral pH (Figure S5c). No activity was detected at pH 8.5 when CO_2_ forms CO_3_^2−^. An acidic condition resulted in decreased activity, presumably because the CO_2_ solubility declines sharply at low pHs.

The IQY was calculated to determine the efficiency of harvesting photons to redox reactions (Figure 2 c). Both junction samples show their highest IQYs at 420 nm, indicating the activity mainly comes from the blue spectral region. CD/FAT exhibits an IQY of 5.9 % at 420 nm, which is 2.8 times that of CD/CN (2.1 %). At longer wavelengths of 500 and 600 nm, CD/FAT still shows activity, and it is a little higher than CD/CN, probably due to the broadened optical window of FAT. As CN could not harvest 500–600 nm photons by itself, the activity at longer wavelengths is due to the light absorption by CD or CD/CN interfacial states. We further measured the quantum efficiency under the light irradiation of a 365 nm LED (please see ESI for details), which was determined as 18.6 %, another remarkable value for a metal‐free system for CO_2_ conversion.

To further confirm the conversion of CO_2_, we have carried out the CO_2_ reduction using ^13^C labelled CO_2_. The peak of ^13^CH_3_OH^+^ (*m*/*z*=33) were observed at *m*/*z*=30, 31, 32 and 33, which were assigned to ^13^CH_3_OH^+^ and three fragments produced during the MS measurement. Such signals are clearly distinguished from those measured in ^12^CO_2_ photoconversion. The evidence indicates that the evolved products originate from the photoreduction of ^13^CO_2_. The stability of a photocatalyst is a crucial factor that determines its long‐term application. Three consecutive runs have also been carried out, and the average rates of methanol production were 1.7, 1.5 and 1.7 μmol h^−1^ for three runs, respectively. The photocatalytic activity of the sample did not show a decrease over 17 hours, indicating the stability (Figure S6a). We also assessed our new junctions’ stability by post‐testing Raman characterisation (Figure S6b). The negligible change was observed before and after reactions, which further proves the stability.

To prove the production of oxygen from water, we also carried out the oxygen production tests on FAT and CD/FAT in the presence of the electron acceptor AgNO_3_ (Figure S6c). Pristine FAT only produced a small amount of oxygen. We attribute the decline in the total amount of oxygen after 1.5 hour is due to the back reaction of oxygen reduction by electrons on Ag metal deposited on the FAT during Ag^+^ reduction reaction and the shielding effect of the metal Ag.^[18]^ The CD/FAT could produce oxygen at a much higher activity compared to FAT alone, in line with improved photocatalytic activity, which is due to the improved charge separation and catalytic effect of CD. Hence, we confirm that FAT and CD/FAT have the thermodynamic driving force to oxidise water and can produce oxygen in the presence of Ag^+^.

Previous theoretical calculations showed that water preferred to adsorb on CD (where holes accumulate). In contrast, methanol favoured adsorption to CN (where electrons accumulate), hence the localisation of the charges was unfavourable for the undesired oxidation of methanol product.^[6a]^ Similarly here, we found that methanol cannot be oxidised on CD/FAT, even in the presence of O_2_ gas, avoiding oxidation of the produced methanol and providing a high selectivity to methanol (Figure 3 a and b). In Figure 3 b, CN is shown to oxidise methanol to CO under the light. After the loading of CD, both the CD/CN and CD/FAT junctions do not show CO production under the light. The fact that CD/CN no longer oxidises methanol supports our interpretation that holes transfer to CD and that holes on CD do not effectively oxidise methanol. Interestingly, pristine FAT polymer does not oxidise methanol to CO on its own, indicating that the surface of FAT does not promote methanol reaction with holes.

Photoluminescence spectroscopy (PL) was undertaken to examine the radiative recombination of the samples (Figure S7). CN exhibits two signals relative to π‐π* and n‐π* transitions. The major emission peaks of FAT are shifted from 500 nm to around 550 nm, and the 450 nm peak decreased due to band gap narrowing, consistent with the previous report.^[10c]^ Although CD shows an emission around 435 nm (Figure S7b), the junctions did not demonstrate enhanced PL signals around 435–450 nm, suggesting that the major absorption comes from the FAT polymer. The signal intensity of CD/FAT is one order of magnitude lower than pure FAT, indicating an enhanced charge separation due to the junction structure.

To understand the origin of the superior performance of CD/FAT photocatalyst, we investigated the electron‐hole dynamics of FAT and CD/FAT aqueous dispersion at μs‐s timescales by TAS using CD/CN as a reference. We observed very broad TAS features, presumably due to a range of energies for trap states.^[19]^ In our previous study on CN, we assigned the TAS signal near 500 nm to holes and signal >600 nm to electrons, supported by the amplitude increase (at 500 nm) and decrease (at >600 nm) in the presence of Ag^+^ ions as electron scavengers (Figure S8a).^[6a]^ Similarly, the photoinduced signal observed in FAT around 550 nm is mainly assigned to photogenerated holes and the broad signal observed around 700–900 nm is assigned to photogenerated electrons (Figure 3 c). The strong peaking near 550 nm at early timescales (<100 μs) is notable and indicative of a higher signal contribution from holes. A quantitative analysis was performed by deconvoluting the experimental spectra to extract the electron and hole signals. The reference electron spectrum was obtained from CD/CN at 100 μs based on 1) the hole‐accepting function of CD and 2) the lack of spectral evolution on longer timescales. The reference hole spectrum was that of CN + 10 mM AgNO_3_ at 100 ms based on 1) the electron‐accepting function of Ag^+^ ions and 2) the decay of the electron signal >600 nm during shorter timescales. These reference spectra were fixed to obtain the best‐fit weight of each component that can reproduce the experimental spectra (Figure S8a). The intensity of the TAS signals of isolated CDs is about 2 orders of magnitude smaller than that of CN and FAT (signals ca. 0.5 %) (Figure S8b). Due to the difference in the intensity, we could not see a TAS signal contribution from CD in the composites and the TAS spectra of CD/CN and CD/FAT resemble that of the CN and FAT rather than the isolated CD.

**Figure 4:**
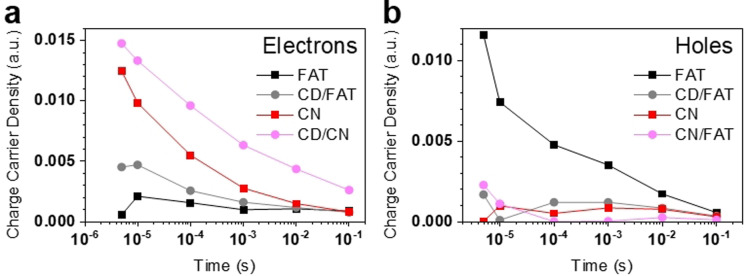
Charge carrier populations of a) electrons and b) holes determined from spectral deconvolution of the TAS spectra for FAT, CD/FAT, CN, and CD/CN samples.

The TA spectra of FAT could be well reproduced using the CN‐derived electron and hole spectra (Figure S9). Spectral deconvolution confirmed that the TA spectrum of CN is dominated by electrons and that of FAT is dominated by holes (Figure 4; also see Figures S10 and S11). Forming the CD/FAT heterojunction notably reduces the intensity of the 550 nm peak (Figure 3 d). This is quantified as an average ca. 3‐fold decreased hole contribution from spectral deconvolution and corresponds to a 75 % hole transfer efficiency to CD. There is an associated average ca. 1.5‐fold increase in the electron contribution. Spectral deconvolution also clearly shows a similar decrease of residual hole signal and increase of electron signal for CD/CN compared to CN. These changes in charge carrier populations are consistent with the hole‐accepting nature of these CD.^[6a]^ The hole‐accepting function of CD leads to enhanced charge separation, which will reduce the rate of recombination, beneficial for photocatalytic activity.

The electrons monitored on the microsecond and longer timescales are trapped in low energy states and do not transfer to CO_2_ since the much more reactive Ag^+^ does not affect the kinetics of the FAT electron decay (Figure S11), consistent with observations with CN.^[9a]^ The similar and slow recombination timescale indicates that the observed holes are also trapped. A lower trapped electron density has previously been linked to improved photocatalytic efficiency for CN.[^9a^, ^12^, ^20^] Considering the initial TAS signal amplitude at 3 μs, the lower electron signal in FAT vs. CN and the improved photocatalytic efficiency of FAT are consistent with these observations and suggest that detrimental electron trapping is much less prominent in FAT. Instead, increased hole trapping occurs in FAT vs. CN. This is in line with the inability of FAT to oxidise methanol whereas CN can drive this photoreaction. As observed in Figure 3 b, even in the presence of O_2_ gas, methanol can not be oxidised by FAT. Taking into account the TAS analysis which shows holes are readily trapped in FAT, it is believed that trapped holes in FAT have a less positive oxidation potential. Therefore, due to both the unfavourable adsorption site on FAT for methanol and the small oxidation potential of the trapped holes in FAT, the methanol produced on CD/FAT can not be oxidised back to CO/CO_2_, resulting in high selectivity to methanol. The detrimental effect on photocatalytic activity of hole trapping is mitigated in the CD/FAT heterojunction since the majority of holes are extracted to the CD on the sub‐microsecond timescale before they can populate trap states (Figure 4 b).

Replacing the linker and terminal N atoms in CN by O atoms in FAT influenced the dynamics of trapped charges. The half‐life time (*t*_50 %_) of the electron signal observed at 800 nm increases by eight folds from 40 μs in CN to 320 μs in FAT (Figure S12). The FAT polymer is thought to be composed of O‐rich and N‐rich domains (Figure 1 b) where the electrons and holes, respectively, tend to separate.[^7b^, ^10c^] We thus attribute the reduced rate of recombination of trapped charges in FAT to the intrinsic spatial separation of electrons and holes and expect that the recombination of shorter‐lived reactive charges will also be reduced.

We do not observe an increased trapped electron lifetime in CD/FAT compared to FAT on the timescales monitored, which is informative considering the changes in charge carrier densities. A four‐fold increase in the electron lifetime was indeed observed for CD/CN vs. CN.^[6a]^ While the origin of the distinct behaviour of FAT and CN when forming a junction with CD is unclear, it is likely that the intrinsic charge separation in FAT mitigates the impact of CD on the recombination of trapped charges. The increased signal of trapped electrons at 3 μs in CD/FAT vs. FAT indicates reduced recombination in the sub‐microsecond timescale, likely relevant to the reactive charges. We, therefore, attribute the improved photocatalytic efficiency in CD/FAT vs. FAT to the interfacial transfer of reactive holes to CD prior to deep trapping and reduced sub‐microsecond charge carrier recombination. The TAS measurements lead us to conclude that holes are the dominant trapped species in FAT while electrons are the dominant trapped species in CN (Figures 4 a and b). When CD extracts holes from FAT, hole accumulation in FAT is not severe in the CD/FAT composite under irradiation and charge recombination remains low. On the other hand, although the CD extracts holes from CN, a higher density of electrons still accumulate in CN when the CD/CN is irradiated and results in higher rates of recombination compared to CD/FAT.

## Conclusion

For the first time, we have demonstrated a unique strategy to improve the performance of CO_2_ reduction to methanol by altering the terminal and linker groups in carbon nitride and forming a junction with hole‐accepting carbon dots. Replacing some N atoms in CN to O atoms in FAT resulted in a lower density of trapped electrons and a higher intensity of trapped holes following photoexcitation, as determined by TAS. Spectroscopic investigations also showed that that CD could extract holes from FAT on the sub‐microsecond timescale, before deep trapping can occur on FAT, to retain the reactivity of holes and increase the number of effective electrons, thus favouring the 6‐electron reduction reaction of CO_2_ fixation to methanol. The CD/FAT exhibited dramatically enhanced visible‐light‐driven methanol production from CO_2_ by water compared to CD/CN. An IQY of 5.9 % was measured at 420 nm for CD/FAT, which is nearly three times higher than that reported for CD/CN. The performance is also found to be dependent on the synthetic microwave power related to the crystallinity of the CD, its loading amount, and the pH. Thus, this work not only paves a sustainable way to close the carbon cycle but also stimulates the photophysical understandings and structural design of polymeric semiconductors.

## Conflict of interest

The authors declare no conflict of interest.

## Supporting information

As a service to our authors and readers, this journal provides supporting information supplied by the authors. Such materials are peer reviewed and may be re‐organized for online delivery, but are not copy‐edited or typeset. Technical support issues arising from supporting information (other than missing files) should be addressed to the authors.

Supporting Information
