# Supplementary material for: Efficient Hole Trapping in Carbon Dot/Oxygen‐Modified Carbon Nitride Heterojunction Photocatalysts for Enhanced Methanol Production from CO2 under Neutral Conditions
Source: Angew Chem Int Ed Engl. 2021 Aug 24;60(38):20811–6. doi: 10.1002/anie.202105570 (PMC8519127; doi:10.1002/anie.202105570)
Supplement: Supplementary file 1 — Supporting Information [file ANIE-60-20811-s001.pdf]

Angewandte Chemie
Eine Zeitschrift der Gesellschaft Deutscher Chemiker

Supporting Information

Efficient Hole Trapping in Carbon Dot/Oxygen-Modified Carbon Nitride Heterojunction Photocatalysts for Enhanced Methanol Production from CO_2 under Neutral Conditions

Yiou Wang, Robert Godin, James R. Durrant, and Junwang Tang**

anie_202105570_sm_miscellaneous_information.pdf

Supporting Information

1. Experimental Section

Materials preparation

All chemical reagents were analytical grade and were used without further purification. Citric acid, N, N-Dimethylformamide (DMF), urea, DCDA (Dicyandiamide), methanol, dichloromethane were purchased from Sigma-Aldrich Company Ltd. Deionised water used in all the experiments had a resistivity of 18.1 MΩ·cm.

Carbon dots (CD) was synthesised from citric acid (3 g, 15.6 mmol) and urea (1 g, 16.7 mmol) dissolved in 8 ml deionised (DI) water. The precursor was treated in the microwave oven for 180 s under 200-300 W. The solid product was dried at 80 ^oC for 10 h, dissolved in water and centrifuged at 8000 r/min for 1 h before extraction by methanol and dichloromethane (v/v = 1:2 and 1:1) and drying to obtain CD powder. The CD samples are noted as CDx, where x stands for the synthetic microwave power. Following the synthesis, we used dialysis bags (1000 Dalton cut-off) to remove potential unreacted species and contaminations and carried out the HRTEM to check the properties of purified CD on FAT polymer.

CN polymer was synthesised as follows: 2 g dicyandiamide in a closed crucible was heated at 500 ^oC for 4 h in static air with a ramp rate of 5 ^oC / min. The yellow solid agglomerates were milled into powders using an agate mortar. 0.5 g of this as-prepared powders were placed on a ceramic plate and heated at 500 ^oC for 4 h in air with a ramp rate of 10 ^oC /min. Then, the CN nanosheets with light yellow colour were finally obtained.

The FAT polymer was synthesised from formic acid (897 μL, 23.8 mmol) and DCDA (2 g, 23.8 mmol) dissolved in 40 mL DI water, as described in our previous report.[1] The solution was set to heat at 130 ^oC for 6 h before drying and calcination in a lidded crucible by a muffle furnace (ramp rate: 2 ^oC / min, 500 ^oC for 4 h).

CD/FAT was synthesized from CD (100-300 mg 5-15% w.t.) and FAT (2 g) suspended in 10 mL DMF. After drying at 60 ^oC for 10 h, the sample was annealed in the same furnace on a ceramic plate (ramp rate: 10 ^oC/ min, 500 ^oC for 4 h), together with pure FAT and CN (to keep consistency). Reference samples involving CD/CN were synthesised from DCDA using the same parameters.[2] DI water, 0.1 M NaOH and HCl were used to wash the produced powders adequately.

Material characterisation

Powder X-Ray Diffraction (PXRD) measurements were taken using a StadiP diffractometer from Stoe company, a voltage of 40 kV, at 30 mA, using a Cu source with $K_{\alpha 1}$ = 1.540562 Å and $K_{\alpha 2}$ = 1.544398 Å. (Company: Stoe. Diffractometer: StadiP. Cu X-ray tube run at 40kV 30mA Capillary transmission geometry. Pre-sample Ge (111) monochromator selects K alpha 1 only. Sample rotated in the beam. Dectris "Mythen 1k" silicon strip detector covering 18 deg 2 theta.) Diffuse reflectance spectra were obtained on a Shimadzu UV-Vis 2550 spectrophotometer fitted with an integrating sphere. A standard barium sulphate powder was used as a reference. Absorption spectra were calculated from the reflection measurements via the Kubelka-Munk transformation. ATR-FTIR spectroscopy was collected using a Perkin-Elmer 1605 FT-IR spectrometer in the wavenumber range 500 – 4000 cm^{-1} with a resolution of 0.5 cm^{-1}. Raman spectroscopic measurements were performed on a Renishaw InVia Raman Microscope, using a 325 nm excitation laser and a wavenumber range 100–2000 cm^{-1}. XPS measurements were obtained on a Thermoscientific XPS K-alpha surface analysis machine using an Al source. The results of etched samples were carried out on the same XPS equipment. The XPS analysis was performed using CasaXPS software. The high-resolution transmission electron microscopic (HR-TEM) images were taken by a Titan Themis at 120 kV accelerating voltage.

Photocatalytic analysis

Before the photocatalytic reduction of CO_2, 10 mg photocatalyst and 10 mL water were added into a septum-sealed borosilicate glass reactor with a volume of 140 mL. Then, the reactor was purged for 20 min with CO_2 before the start of the photoreduction experiment. A 300 W Xe lamp (Newport) was utilised as a light source, and the light output power was measured by a Newport 918-D calibrated photodetector. During the reaction, the products were analysed by GC (Varian GC-450) with a thermal conductivity detector (TCD, connected to a molecular sieve column) and a flame ionisation detector (FID, connected to a CP-SIL 5CB capillary column) containing a methanizer equipment. Ar gas was used as the GC carrier gas. The CH_3OH oxidation conditions:

10 ml H_2O, 0.12 µmol MeOH, 10 mg CD/FAT photocatalyst, 300 W Xenon lamp irradiation with 420 nm long-pass filter in 1 bar Argon atmosphere.

Calculation of internal quantum efficiency

The internal quantum yields for CD/FAT photocatalyst was measured using the same experimental setup as the photocatalysis measurement, with a bandpass filter (λ=420 nm, 500 nm or 600 nm).

The internal quantum yields are defined by the following equation:

$$IQY = \frac{number\ of\ reacted\ electrons}{number\ of\ the\ absorbed\ photons} \times 100\% \qquad (3)$$

Two electrons are consumed by per CO molecule evolved, and six electrons are consumed by per CH_3OH molecule evolved according to reaction (3) or (4).

$$CO_2 + 2e^- + 2H^+ \rightarrow CO + H_2O \qquad (4)$$

$$CO_2 + 6e^- + 6H^+ \rightarrow CH_3OH + H_2O \qquad (5)$$

As a result, the internal quantum efficiency can be estimated by the equation:

$$IQY = \frac{N_{CO} \times 2 \times N_A + N_{MeOH} \times 6 \times N_A}{H_a \times A \times \frac{\lambda}{hc} \times t} \qquad (6)$$

where: N_{CO} is the amount of CO, N_{MeOH} is the amount of CH_3OH, N_A is the Avogadro's number, H_a is the average intensity of absorbed light, obtained by the subtraction of the transmitted intensity from the incident intensity. A is the irradiation area (12 cm^2), *h* is the Planck's constant, c is the speed of light, λ is the wavelength of the incident light, t is the time.

The quantum yield report here is the internal quantum yield, which was calculated based on the absorbed light density (by measurement of the light intensities in front of and behind the reactor containing 10 mg CD/FAT in 10 ml water). For example, the absorbed light intensity at 420 nm was 161.2 $\mu W/cm^2$, and 0.242 µmol methanol was collected in 1 hour (3600 s). No CO was produced which leads to $N_{CO} = 0$.

$$IQY = \frac{N_{CO} \times 2 \times N_A + N_{MeOH} \times 6 \times N_A}{H_a \times A \times \frac{\lambda}{hc} \times t} = \frac{0.242\ \mu mol \times 6 \times 6.02 \times 10^{23} mol^{-1} \times 6.626 \times 10^{-34} Js \times 3 \times\ 10^{8} ms^{-1}}{161.2 \mu W cm^{-2} \times 12 cm^{2} \times 420 \times 10^{-9} \times 3600 s} = 5.9\% \qquad (7)$$

We are also interested in the activity at a short wavelength and have measured the quantum efficiency under the light irradiation of a 365 nm LED (Thorlabs M365LP1). In 5 hours, 7.15 micromoles of methanol have been produced and we calculate an internal quantum efficiency of 18.6%, another remarkable value for a metal-free system.

$$IQY = \frac{N_{CO} \times 2 \times N_A + N_{MeOH} \times 6 \times N_A}{H_a \times A \times \frac{\lambda}{hc} \times t} = \frac{7.15\ \mu mol \times 6 \times 6.02 \times 10^{23} mol^{-1} \times 6.626 \times 10^{-34} Js \times 3 \times\ 10^{8} ms^{-1}}{2100 \mu W cm^{-2} \times 2 cm^{2} \times 365 \times 10^{-9} \times 3600 s/hour \times 5 hour} = 18.6\% \qquad (6)$$

Transient absorption spectroscopy

TAS data were acquired on home-built setups as described previously.[3] Samples were purged with argon to remove oxygen. 355 nm or 600 nm laser excitation was generated from an Nd:YAG laser (OPOTEK Opolette 355 II, 7 ns pulse width). The 355 nm excitation fluence was set to 460 $\mu J/cm^2$ The probe light was generated from a quartz halogen lamp (Bentham IL1). Long pass filters (Comar Instruments) were placed between the lamp and sample to minimise short-wavelength irradiation of the sample. A 5 cm path length cuvette filled with DI water was also placed in the beam path as an IR filter to avoid heating effects. A long pass filter positioned between the sample and a monochromator was used to block the scattered laser light.

The probe wavelength was selected by the monochromator, and the light relayed to a Si photodiode detector (Hamamatsu S3071). Data on the sub-ms timescale were conditioned by an electronic amplifier box (Costronics) and recorded on an oscilloscope. Data on the ms timescale were simultaneously recorded on a National Instruments DAQ card. Acquisitions were triggered by a photodiode (Thorlabs DET10A) exposed to laser scatter. Data from at least 32 laser pulses were acquired and processed using software written in the Labview environment (Austin Consultants) to obtain kinetic traces. The linearly spaced data (ca. 35k points) was reduced to logarithmically spaced data (ca. 160 points) with averaging to reduce the noise.

Reconstructed spectra [%Abs(λ)] were generated by multiplying the weight of the electron and hole components ($W_{electron}$, W_{hole}) with the fixed reference spectra shown in Figure S7. Mathematically, this can be written as:

$$\%Abs(\lambda) = W_{electron} \times Spectrum_{electron}(\lambda) + W_{hole} \times Spectrum_{hole}(\lambda)$$

The best fit was found using the non-linear least square solver lsqcurvefit from MATLAB to minimise the difference between the experimental and reconstructed spectra.

Table S1. A literature review of CO_2 reduction to MeOH under visible light in aqueous environments with reported quantum yields. The work represents a record quantum yield and is much better than any metal-free/polymer photocatalysts, even better than the best inorganic photocatalyst $BiVO_4$.

	Photocatalyst	IQY@420nm if not specified	O_2 gas measurement	Selectivity/%	Reference
1	CD/FAT	5.9	√	~100	This work
2	BiVO4	4.5 (apparent QY)	√	~95	[4]
3	CISCN20	0.14 (apparent QY)	×		[5]
4	^{m}CD/CN	2.1	√	99.6	[3]
5	anatase–brookite TiO_2 composite	0.0717	×		[4, 6]
6	NiO/InTaO4	0.063	×		[5, 7]
7	mesoporous flake g-C_3N_4	0.18	×	58.3	[3, 8]
8	rGO–CuO	1.3 (visible LED)	√		[6, 9]
9	graphene− TiO_2	2.3 (lamp in the middle of the reactor)	×		[7, 10]
10	$BiVO_4$ (0.2g)	0.22 (visible)<br>0.24 (full sepectrum)	√		[8, 11]
11	Carbon nitride-CdS QD	0.91 @ 435 nm (apparent QY)	×	73	[9, 12]
12	$In_2O_{3-x}(OH)_y$	0.19@250ºC	×	50	[10, 13]

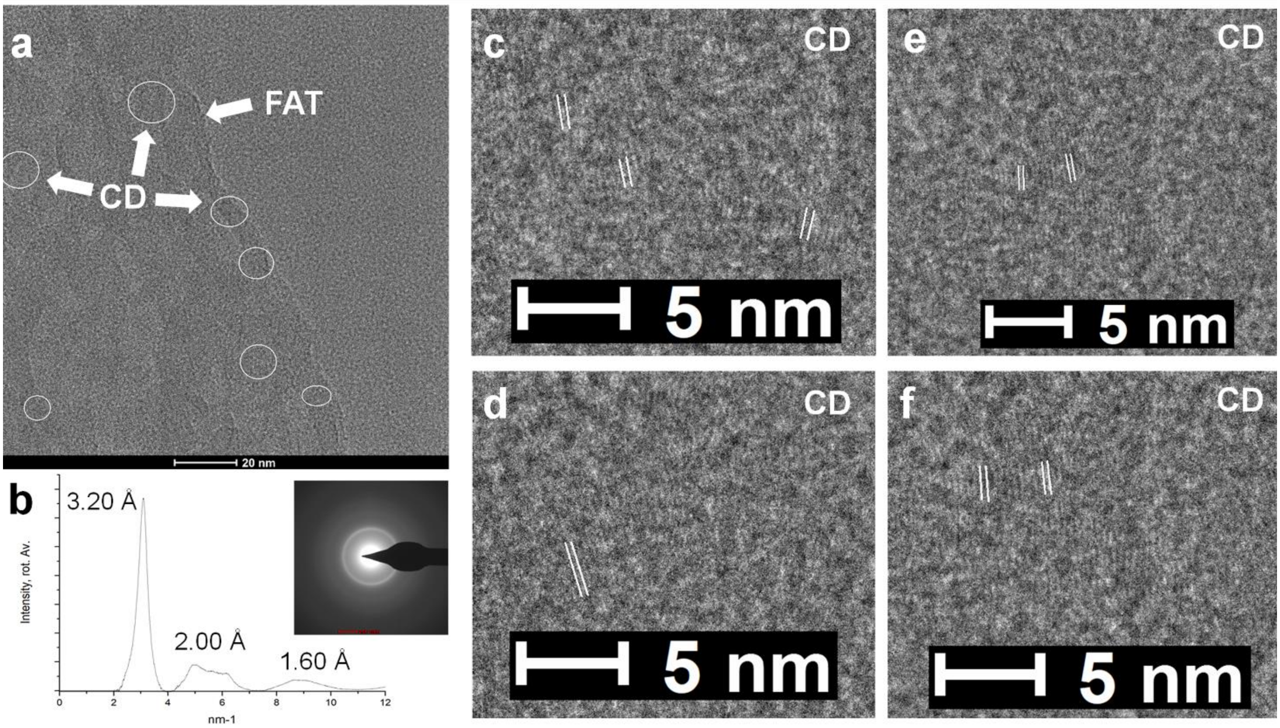

Figure S1. (a) HRTEM image of CD in CD/FAT polymer nanocomposite. CD has been marked by circles. Scale bar: 20 nm. (b) FFT patterns of D spacing. (c)-(f) Enlarged images showing graphite superstructure of CD. Scale bar: 5 nm.

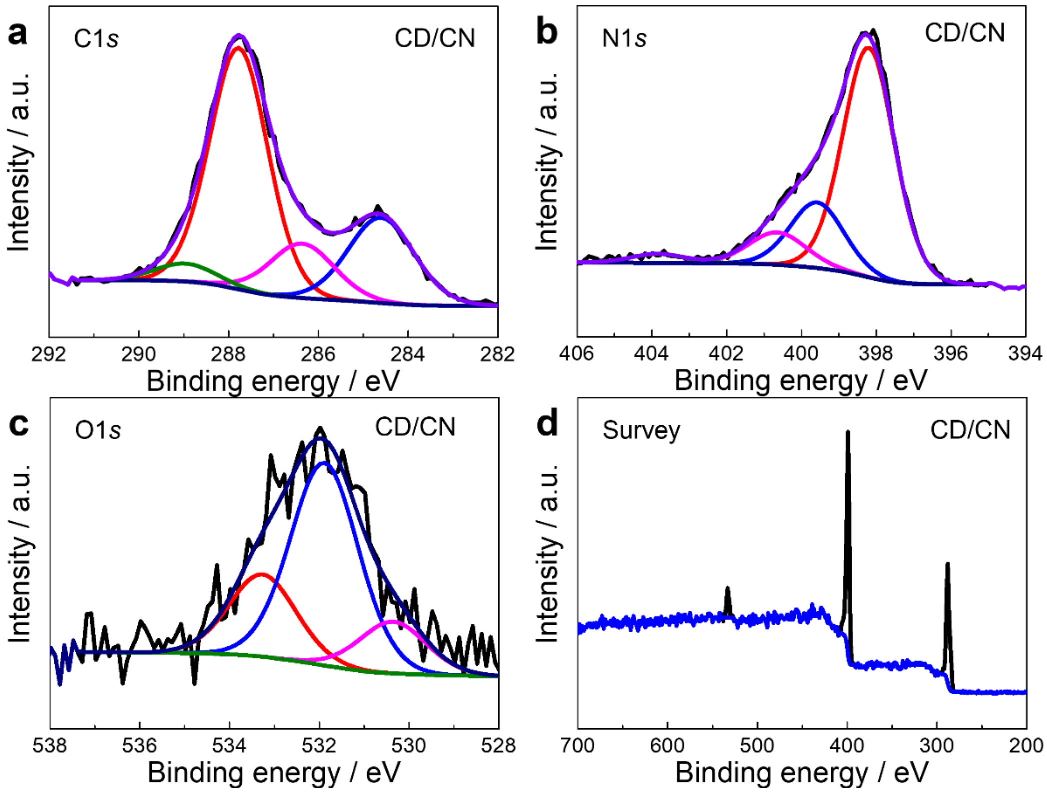

Figure S2. C1s (a), N1s (b), O1s (c) and survey (d) XPS spectra of CD/CN sample.

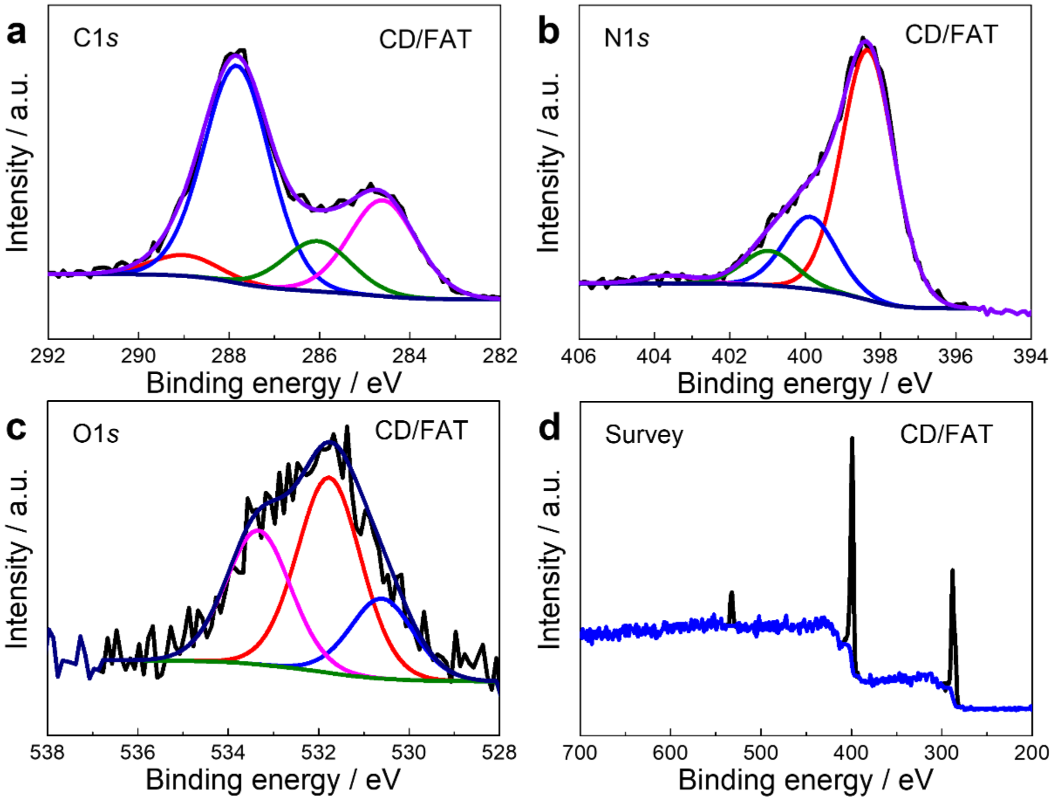

Figure S3. C1s (a), N1s (b), O1s (c) and survey (d) XPS spectra of CD/FAT sample.

Table S 2 Surface compositions of C, N, O from XPS spectra for samples used in this study

Sample	C (at %)	N (at %)	O (at %)
CD (herein)	61.99	11.86	26.15
CN	48.41	49.26	2.33
FAT	50.30	42.59	7.11
CD/CN	49.19	47.17	3.64
CD/FAT	51.10	45.09	3.81

Table S 3 Compositions of C 1s XPS spectra for samples used in this study[14]

Sample	C=O (289 eV)	C-N (287.5 eV)	C-O (286.2 eV)	C-C (284.6 eV)
CD	22.32	25.05	10.55	42.08
FAT	-	62.09	8.51	29.40
CN	-	61.83	3.67	34.51
CD/FAT	-	60.06	13.70	26.24
CD/CN	-	65.39	11.35	23.26

Table S 4 Compositions of N 1s XPS spectra for samples used in this study[14b]

Sample	NH_x (401 eV)	$N-C_3$ (400 eV)	C=N-C (398.7 eV)
CD	33.14	51.18	15.68
FAT	6.74	45.05	50.20
CN	9.31	26.41	64.27
CD/FAT	14.39	20.26	65.35
CD/CN	16.27	26.47	57.25

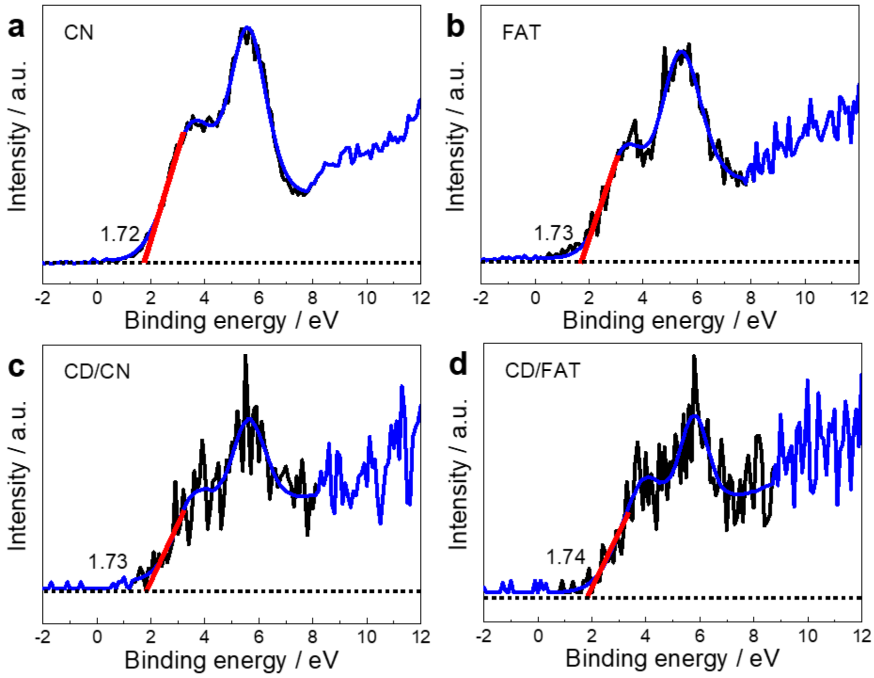

Figure S4. (a)-(d) Valence band XPS of CN, FAT, CD/CN, CD/FAT.

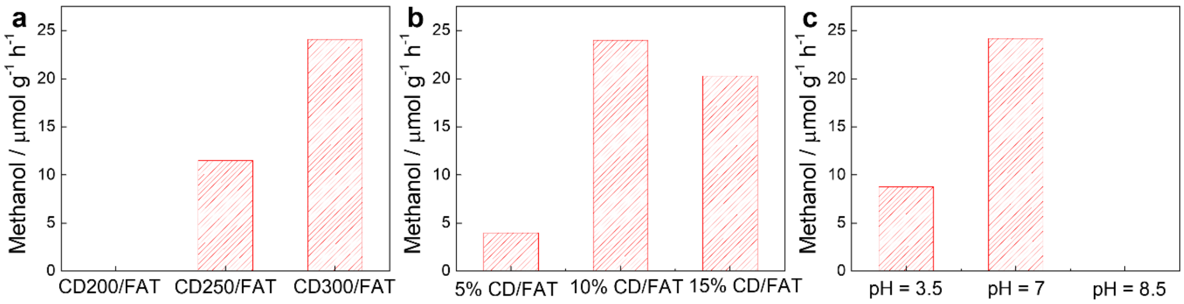

Figure S5. Photocatalytic CO_2 reduction to methanol (a) with CD synthesized under different microwave power, (b) with various loading amount of CD on FAT and (c) at different pHs during reaction.

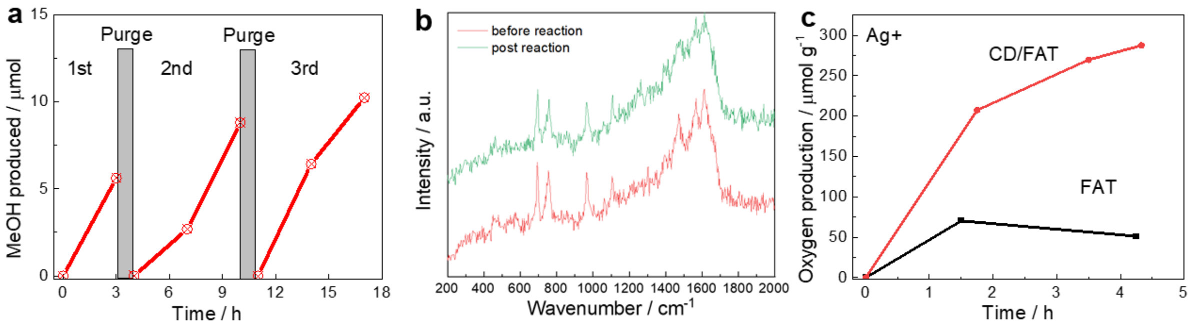

Figure S6. (a) Consecutive three runs of CO_2 photoconversion to methanol on CD/FAT. Conditions: 10 mg CD/FAT was dispersed in 2 mL water in a 10 mL reactor. The system was purged by CO_2 for 5 min prior to the irradiation of a 450 W Xenon lamp before each run. (b) Raman spectra of CD/FAT before and after photocatalytic reaction (c). water oxidation measured on FAT and CD/FAT in the presence of 0.1 M aqueous $AgNO_3$. Conditions: 10 mg CD/FAT or FAT was dispersed in 2 mL 0.1M $AgNO_3$ solution in a 10 mL reactor. The system was purged by Argon for 5 min prior to the irradiation of a 450 W Xenon lamp.

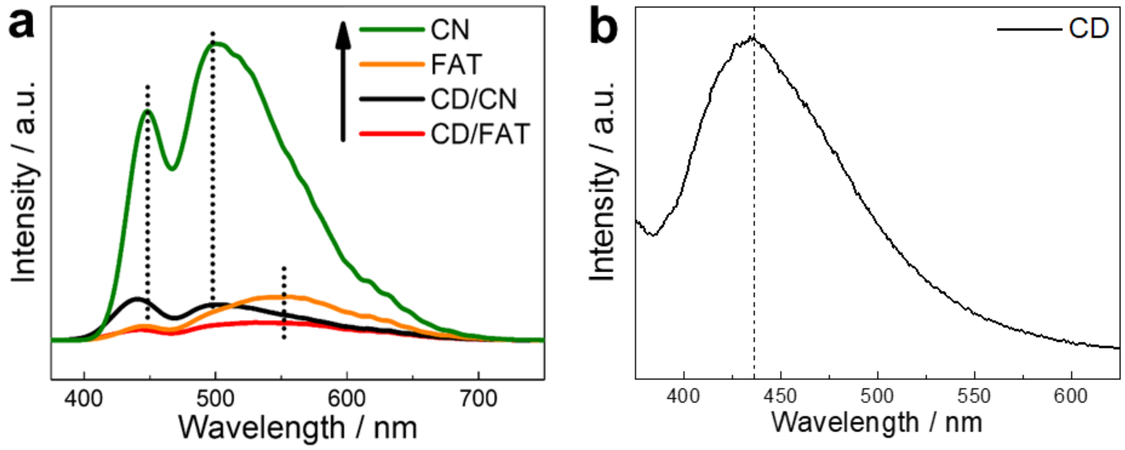

Figure S7. (a) photoluminescence spectra (325 nm laser excitation) of CN, CD/CN, FAT and CD/FAT. (b) photoluminescence spectra (325 nm laser excitation) of CD in water.

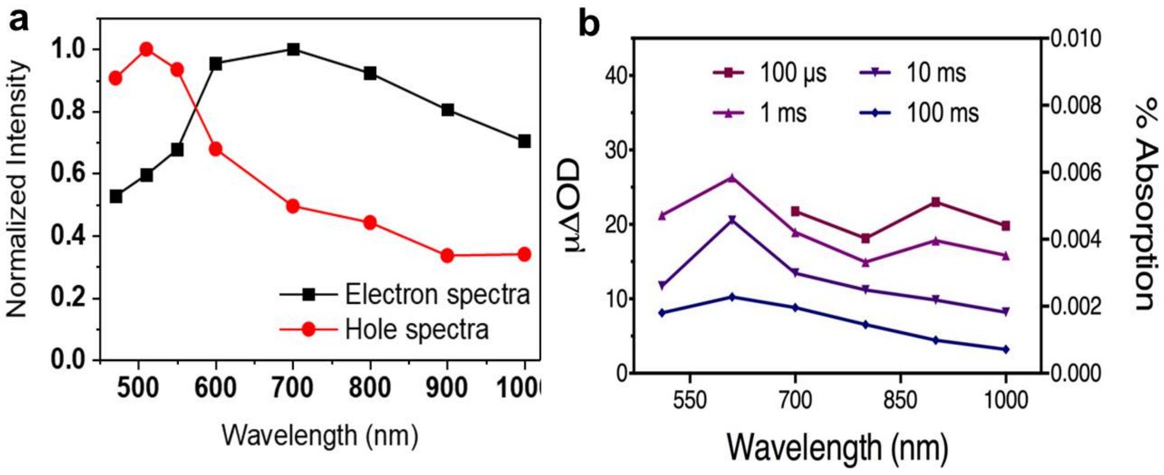

Figure S8. (a) Reference TAS spectra for electron and hole used in spectral deconvolution. The electron spectrum is taken from CD/CN at 100 µs, consistent with the hole-accepting function of CD. The hole spectrum is taken from CN + 10 mM $AgNO_3$, a strong electron scavenger, at 100 ms. (b) Transmission TAS spectra of CD (0.6 mg/mL) in aqueous solution.

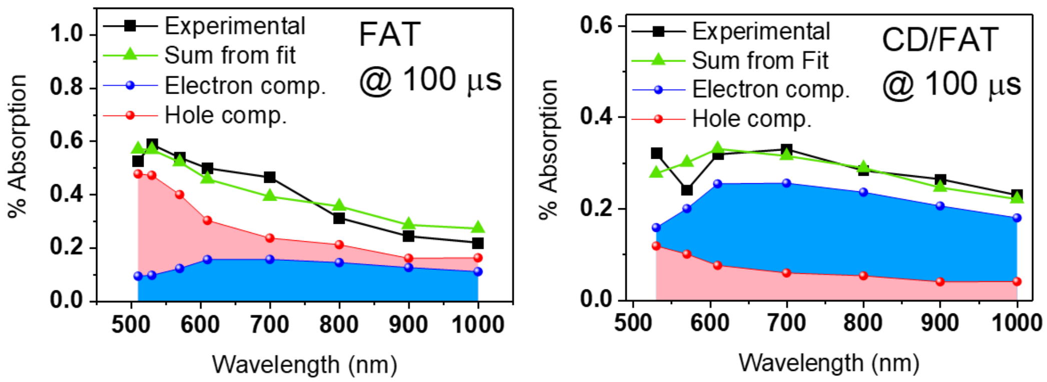

Figure S9. Example of the spectral deconvolution results for FAT (left) and CD/FAT (right) spectra at 100 µs. The electron contribution is shown in blue and the hole contribution is shown in red. The sum of both is shown at the green line and compared with the experimental results shown as the black line.

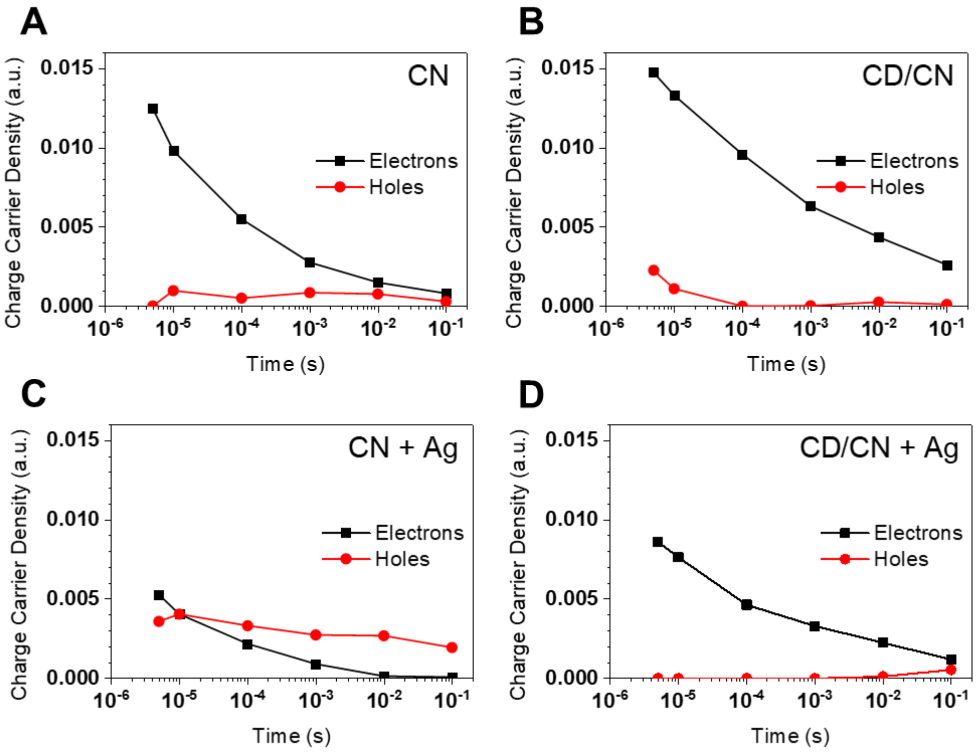

Figure S10. Deconvoluted electron and hole population for the CN sample series. The Y axis values are the best-fit weight of the normalized spectra shown in Figure S7. 10 mM $AgNO_3$ was used as an electron scavenger in C) and D).

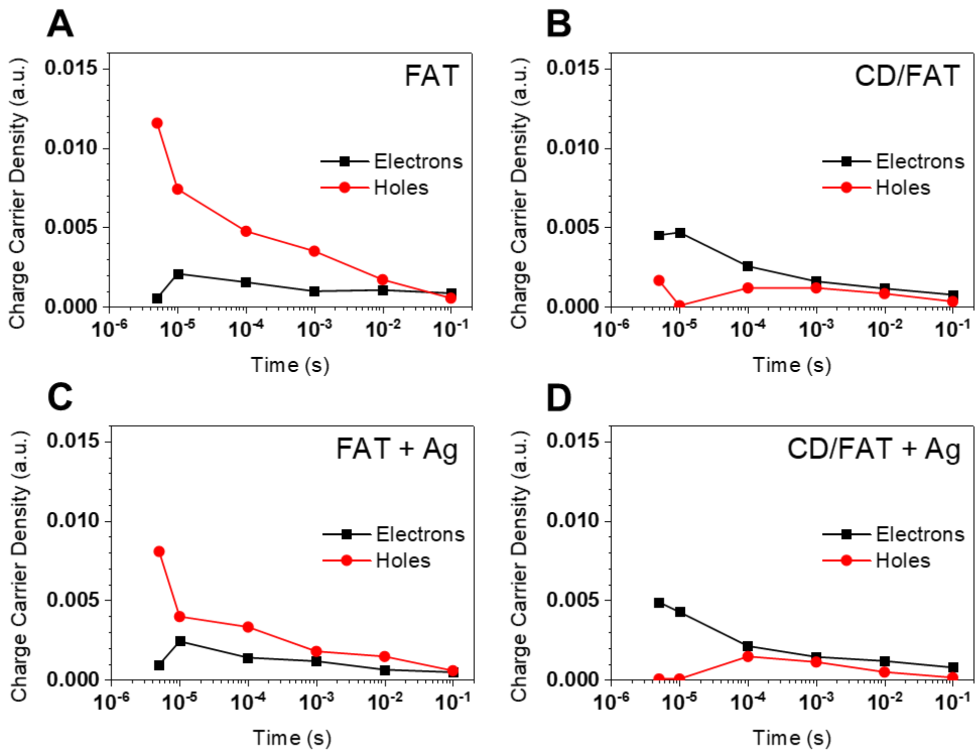

Figure S11. Deconvoluted electron and hole population for the FAT sample series. The Y axis values are the best-fit weight of the normalized spectra shown in Figure S7. 10 mM $AgNO_3$ was used as an electron scavenger in C) and D). In contrast to CN, the addition of Ag^+ did not affect the TAS signals for the FAT samples. We ascribe this effect to the lower CB edge of FAT compared to CN which can lower the driving force for electron transfer of trapped electrons with microsecond or longer lifetimes.

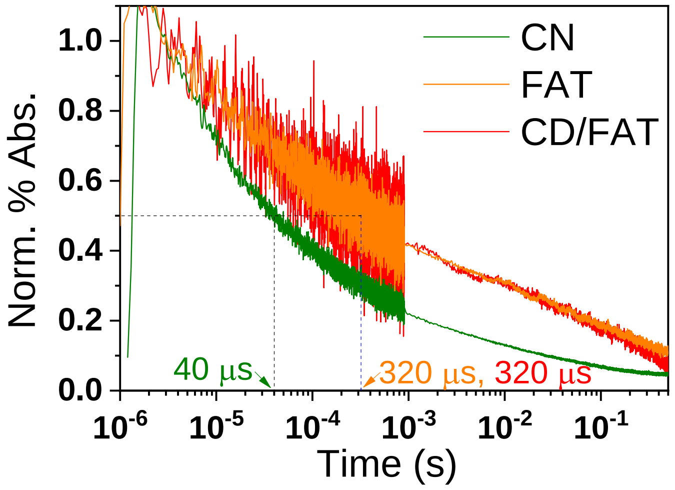

Figure S12. μs-TAS decay kinetics normalized at 3 μs of CN, FAT and CD/FAT in water monitored at 800 nm and excited by pulsed 355 nm excitation (460 μJ/cm^2).

References:

[1] Y. Wang, F. Silveri, M. K. Bayazit, Q. Ruan, Y. Li, J. Xie, C. R. A. Catlow, J. Tang, *Advanced Energy Materials* **2018**, *0*, 1801084.

[2] Y. Di, X. Wang, A. Thomas, M. Antonietti, *ChemCatChem* **2010**, *2*, 834-838.

[3] Y. Wang, X. Liu, X. Han, R. Godin, J. Chen, W. Zhou, C. Jiang, J. F. Thompson, K. B. Mustafa, S. A. Shevlin, J. R. Durrant, Z. Guo, J. Tang, *Nat. Comm.* **2020**, *11*, 2531.

[4] S. Gao, B. Gu, X. Jiao, Y. Sun, X. Zu, F. Yang, W. Zhu, C. Wang, Z. Feng, B. Ye, Y. Xie, *Journal of the American Chemical Society* **2017**, *139*, 3438-3445.

[5] H. Liu, Z. Zhang, J. Meng, J. Zhang, *Molecular Catalysis* **2017**, *430*, 9-19.

[6] Q. D. Truong, T. H. Le, J.-Y. Liu, C.-C. Chung, Y.-C. Ling, *Appl. Catal., A* **2012**, *437*, 28-35.

[7] Z.-Y. Wang, H.-C. Chou, J. C. S. Wu, D. P. Tsai, G. Mul, *Appl. Catal., A* **2010**, *380*, 172-177.

[8] J. Mao, T. Peng, X. Zhang, K. Li, L. Ye, L. Zan, *Catal. Sci. Technol.* **2013**, *3*, 1253-1260.

[9] R. Gusain, P. Kumar, O. P. Sharma, S. L. Jain, O. P. Khatri, *Appl. Catal., B* **2016**, *181*, 352-362.

[10] Q. Zhang, C.-F. Lin, B.-Y. Chen, T. Ouyang, C.-T. Chang, *Environmental Science & Technology* **2015**, *49*, 2405-2417.

[11] J. Mao, T. Peng, X. Zhang, K. Li, L. Zan, *Catal. Commun.* **2012**, *28*, 38-41.

[12] A. Li, T. Wang, C. Li, Z. Huang, Z. Luo, J. Gong, *Angew. Chem. Int. Ed.* **2019**, *131*, 3844-3848.

[13] L. Wang, M. Ghoussoub, H. Wang, Y. Shao, W. Sun, A. A. Tountas, T. E. Wood, H. Li, J. Y. Y. Loh, Y. Dong, M. Xia, Y. Li, S. Wang, J. Jia, C. Qiu, C. Qian, N. P. Kherani, L. He, X. Zhang, G. A. Ozin, *Joule* **2018**, *2*, 1369-1381.

[14] a) B. Ozturk, A. de-Luna-Bugallo, E. Panaitescu, A. N. Chiaramonti, F. Liu, A. Vargas, X. Jiang, N. Kharche, O. Yavuzcetin, M. Alnaji, M. J. Ford, J. Lok, Y. Zhao, N. King, N. K. Dhar, M. Dubey, S. K. Nayak, S. Sridhar, S. Kar, *Science Advances* **2015**, *1*; b) D. J. Martin, K. Qiu, S. A. Shevlin, A. D. Handoko, X. Chen, Z. Guo, J. Tang, *Angew Chem Int Edit* **2014**, *53*, 9240-9245.
